# The intrinsic piezoelectricity of monoclinic Pb(Zr_1−*x*_Ti_*x*_)O_3_†

**DOI:** 10.1039/d4ra06270a

**Published:** 2024-12-02

**Authors:** Junyu Niu, Chong Li, Zengzhe Xi

**Affiliations:** a Shaanxi Key Laboratory of Photoelectric Functional Materials and Devices, School of Materials and Chemical Engineering, Xi'an Technological University Xi'an 710021 Shaanxi China zzhxi@xatu.edu.cn; b Shenzhen Key Laboratory of Advanced Thin Films and Applications, Key Laboratory of Optoelectronic Devices and Systems of Ministry of Education and Guangdong Province, College of Physics and Optoelectronic Engineering, Shenzhen University Shenzhen 518060 P. R. China

## Abstract

The phase diagram of Pb(Zr_1−*x*_Ti_*x*_)O_3_ (PZT) is quite clear; however, the existence and intrinsic piezoelectricity of low-symmetry structures near the MPB (morphotropic phase boundary) remain subjects of considerable uncertainty. The impact of the MPB on the piezoelectric properties of PZT was investigated *via* first principles study. Different PZT structures were established by virtual crystal approximation (VCA). The density functional theory (DFT) with plane-wave basis set method was utilized to calculate the energy, elastic, and piezoelectric properties. The presence of the monoclinic phase in MPB was confirmed near *x* ∼ 0.53. This phase exhibited distinct piezoelectric properties. Although the coefficients *d*_33_ and *d*_15_ were lower, the maximum value of *d*_31_ reached −198.33 pm V^−1^. By exhaustively exploring all possible structures at the MPB, we demonstrate that the intrinsic piezoelectricity of monoclinic structures is not the main contributor to the MPB effect. Further study shows that this is related to the hybridization between the O2p orbital and the d orbital of the central atom. Finally, analysis of the energy barrier along the polarization rotation paths implies a flattening of free energy in the monoclinic phase, suggesting a high intrinsic piezoelectric coefficient. The intrinsic piezoelectric properties of the monoclinic phase may bring new ideas to the study of the MPB effect.

## Abstract 2

The *Cm*(M_B_) structure of PZT was confirmed by DFT as a stable phase *via* phonon and covalent bonding analysis. Given its lower piezoelectric, the intrinsic piezoelectricity of low-symmetry phase was confirmed not the major contributor to MPB effect.

## Graphical abstract

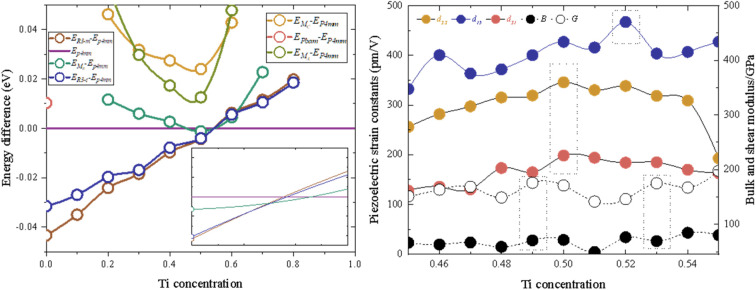

## 1. Introduction

Lead-based piezoelectric materials have garnered significant attention owing to their exceptional ferroelectric properties.^1–7^ PZT, as a perovskite-type ferroelectric material, exhibits different phases near Curie temperature (*T*_c_).^8^ Above *T*_c_, PZT crystals exist in a cubic phase. Below *T*_c_, PZT consists of a continuous solid solution of PbTiO_3_ and PbZrO_3_. At Ti concentration greater than 0.53, the microstructure primarily consists of the tetragonal phase. Conversely, at Ti concentration of less than 0.53, the microstructure consists of the rhombohedral phase. Currently, the most widely used PZT material is located at the MPB.^1,2,6,9,10^

There is no consensus on the MPB effect, although there are two main conjectures regarding this effect. The first theory is “field-induced phase transition”. Shrout *et al.* found that applying a high electric field in the [001] direction induces a transformation from the rhombohedral to the tetragonal phase, accompanied with a large strain in relaxor single crystals.^11^ Another theory is polarization rotation, first proposed by Fu *et al.*^12^ They compared the energy of different polarization rotation paths in BaTiO_3_. Noheda *et al.* proved the polarization rotation path along a–g–d–e in Pb(ZnNb)O_3_–PbTiO_3_ single crystals.^13^ The flattening of the free energy surface was considered a major factor contributing to high piezoelectricity. At present, polarization rotation theory is recognized by a large number of researchers.^14,15^ However, the intricacies of the MPB exceed conventional understanding. Even pure PbTiO_3_, which is usually considered unrelated to the MPB, demonstrated an MPB containing two monoclinic structures under a certain pressure in theory.^16^

In recent years, beyond the study of domain wall structures,^17–21^ researchers have focused on the contribution of intrinsic piezoelectricity of the low symmetry phase, especially the monoclinic phase.^22–30^ Bai *et al.* proved that the shear strain of a monoclinic structure has an important influence on its ferroelectric properties through thermodynamics.^31^ Damjanovic emphasized the crucial role played by intrinsic piezoelectricity in the MPB.^32^ The monoclinic phase, serving as a structural entity along the phase transition path, constitutes a significant subject for investigating lead-based piezoelectric crystals through the polarization rotation theory. In 1999, Noheda *et al.* confirmed that the monoclinic PZT brings unique ferroelectric mechanisms.^33^ Cohen *et al.* confirmed the existence of the R–M_A_–T transition utilizing DFT.^12^ In 2016, the diffraction peak of a certain *Cm* structure in PZT was found *via* SXRD.^34^ Neutron diffraction also confirmed the existence of low-temperature *Cc* structure and high-temperature *Cm* structure in PZT.^35,36^ However, the academic community still lacks a complete understanding of the stability and intrinsic piezoelectric properties of various monoclinic phases. Liu *et al.* believed that compared with other structures, the M_A_ structure is the key factor in generating the giant piezoelectric response of a perovskite crystal.^37,38^ On the phase boundaries of the two monoclinic structures, the larger displacement of Pb in the M_A_ structure in the external field or strain might be more advantageous in piezoelectric response.^39^ Jin *et al.* proposed the ferroelectric adaptive theory, which suggests that the monoclinic phase is composed of rhombohedral phase or tetragonal phase nano-twins, which appear as monoclinic phase as a whole, and such diffraction spots have been observed in Pb(MnNb)O_3_–PbTiO_3_ ceramics and Pb(ZnNb)O_3_–PbTiO_3_ single crystals. The emergence of these studies raised new doubts about the intrinsic piezoelectric properties of the monoclinic phase.^40,41^ However, evidence was found that the pure *Cm* structure existed on the side of the tetragonal phase, not the adaptive phase in the PZT, by convergent beam electron diffraction in 2011.^4^

Additionally, DFT is considered to be an effective method to study piezoelectric effects.^42,43^ Bellaiche *et al.* used the DFT method to study the properties of PZT in a certain temperature range at MPB and proposed the possibility of a low-temperature monoclinic phase as a polarization rotation mesophase in 2000.^44^ Liu *et al.* determined the position of MPB of PZT. Furthermore, the piezoelectric and elastic properties can be calculated.^45^ Duerloo *et al.* predicted four novel high piezoelectric materials using this DFT method.^46^ Ghosez *et al.* evaluated the differences in the crystal dynamics of the BaTiO_3_, PbTiO_3_, and PbZrO_3_ lattices *via* the calculation of phonon scattering.^47^

Finally, monoclinic structure as the transition state of ferroelectric phase transition is a crucial part of polarization rotation theory. The intrinsic piezoelectricity of the monoclinic phase is a significant aspect for researchers to understand the MPB effect. Therefore, in this study, we determined the existence and intrinsic piezoelectricity of monoclinic phases by first principles calculations in detail.

## 2. Computational methods

This study employed the open-source ABINIT software. For the purpose of investigating the intrinsic piezoelectric effect in different 2 × 2 × 2 VCA lattices, the exchange-correlation potential for electrons was described using GGA implemented in the PBE scheme.^48^ The Monkhorst–Pack method was employed for sampling the *K* point in the Brillouin zone, with a grid density of 6 × 6 × 6. The BFGS algorithm was employed for geometry optimization during the calculations. The cutoff energy was set to 600 eV, and the maximum displacement was 0.0005 Å. To enhance accuracy, the convergence for the maximum tolerance on the plane wave function squared residual was 10^−18^. The maximum force tolerance was 0.01 eV Å^−1^. The electric polarizations were calculated using the Berry phase method.^49^

## 3. Results and discussion

**Fig. 1:**
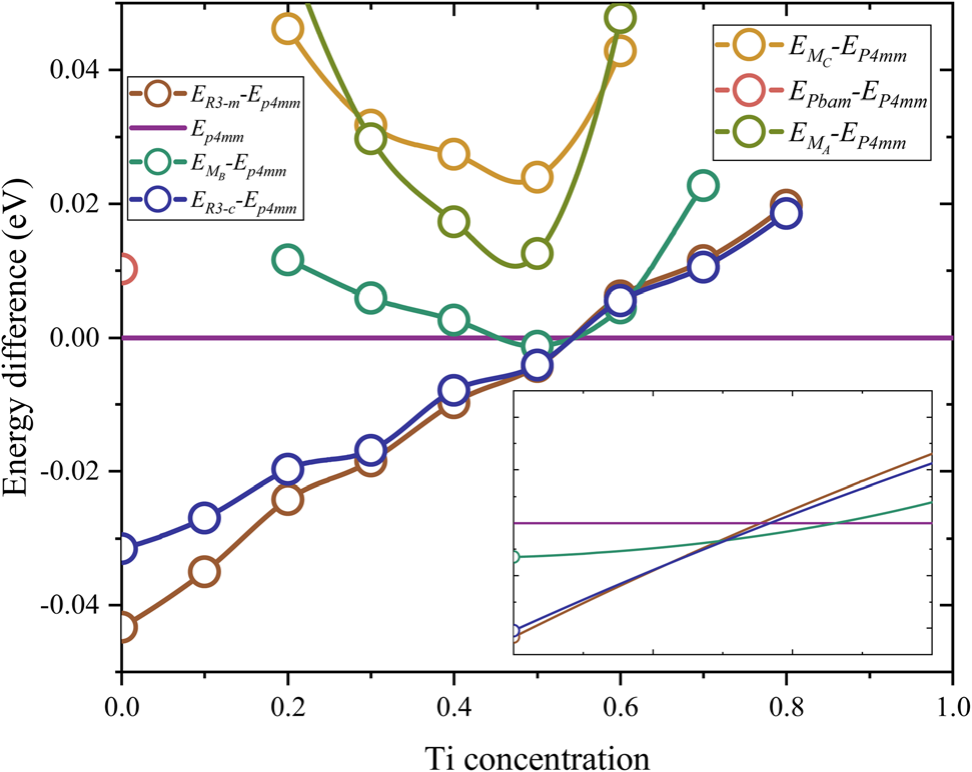
The difference in total energy between *P*4*mm* and other phases. The spectral line closest to the top in the figure will have the lowest total energy to obtain a stable structure.

Fig. 1 illustrates the total energy differences between different phases to *P*4*mm*. Table S1† shows the lattice parameters of these structures and Fig. S1† shows the total energy of this phase. The energy difference is defined *via* the formula *E*_D_(*x*) = *E*_*P*4*mm*_(*x*) − *E*_phase_(*x*). Firstly, in the Zr-rich region (*x* < 0.53), the *R*3̄*m* phase exhibits the lowest total energy compared to other phases, with a maximum difference of around 0.04 eV. In the Ti-rich region (*x* > 0.53), the *P*4*mm* phase shows the lowest total energy, which indicates stability in this range. Secondly, high Ti content is not conducive to the existence of a rhombohedral phase; when *x* > 0.8, the total energy of *R*3̄*m* and *R*3̄*c* structure will fail to converge. Furthermore, at lower Ti concentration, the *R*3̄*m* phase exhibits lower total energy compared to the *R*3̄*c* phase. Similarly, this phenomenon is also reflected in the three monoclinic structures; in the range of *x* < 0.2 and *x* > 0.7, their structures also fail to converge. It is noteworthy that the *Cm*(M_B_) phase has higher total energy at any Ti concentration, except for a local extremum near *x* = 0.51, which closely aligns with MPB (*x* ∼ 0.53). Additionally, the total energy and convergence are not the only verification methods for the existence. In view of this, the study attempts to calculate the free energy to study the existence of *Cm*(M_B_) structures. For other monoclinic phases (M_C_, M_A_), they exhibit an excessive total energy at the MPB, with a significant energy barrier to even the nearest stable M_B_ structures (0.012 eV, 0.023 eV).

By calculating the phonon scattering situation at the *Γ* point, the relationship between energy and temperature can be determined, expressed as:1
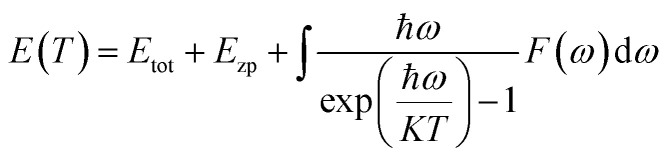


The zero-point energy is:2
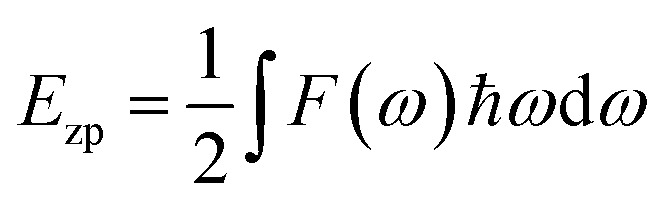


While the free energy *F* can be expressed as:3
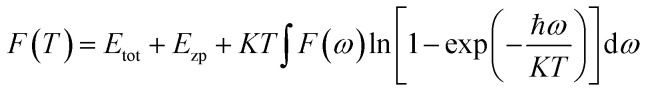


At 0 K, the free energy is equal to the zero-point energy.

**Fig. 2:**
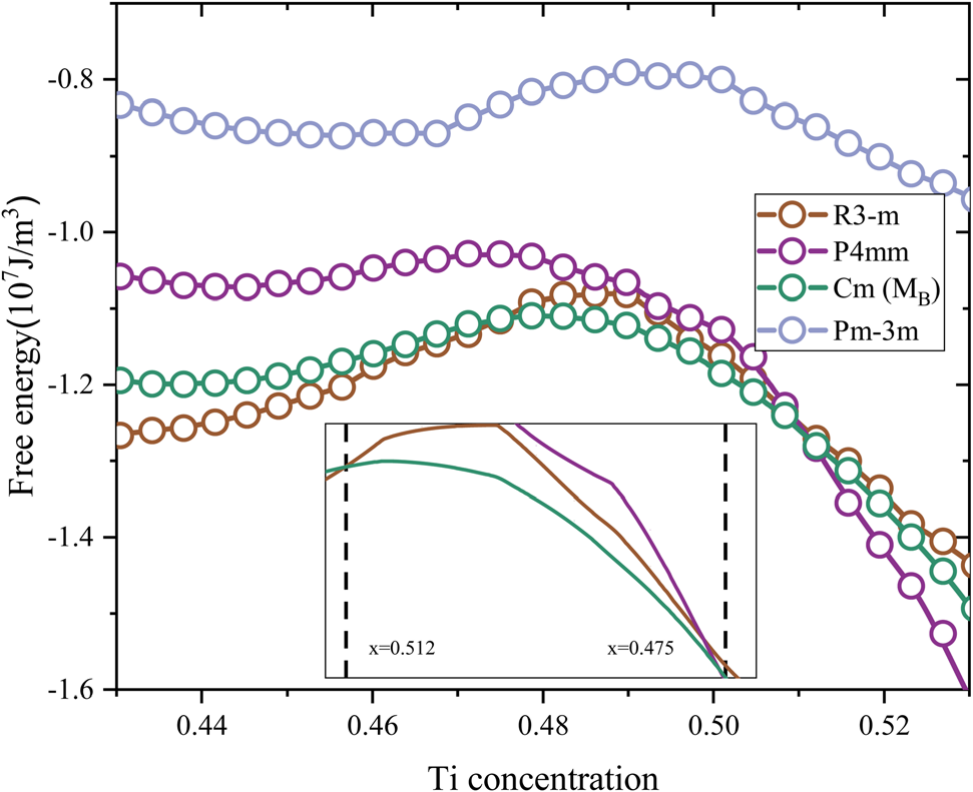
The free energy near the MPB. The detailed scope is shown in the accompanying figure.

Based on the calculations of the free energy in Fig. 2, the *P*4*mm* phase has the lowest free energy for *x* > 0.512, while the *R*3̄*m* phase has the lowest free energy for *x* < 0.475.^50^ The range in which the free energy of both takes its minimum is very close to the range commonly considered for the MPB of the PZT. Within a narrow range of 0.475 < *x* < 0.512, the *Cm*(M_B_) phase exhibits the lowest free energy, indicating its stable existence in this chemical composition. These two methods (total energy and free energy) yield very close results. In order to further explore the origin of this stability of *Cm*(M_B_), the paper first selects the structure of *x* = 0.5 as the research object. Subsequently, phonon scattering spectra are calculated *via* the linear response method.

**Fig. 3:**
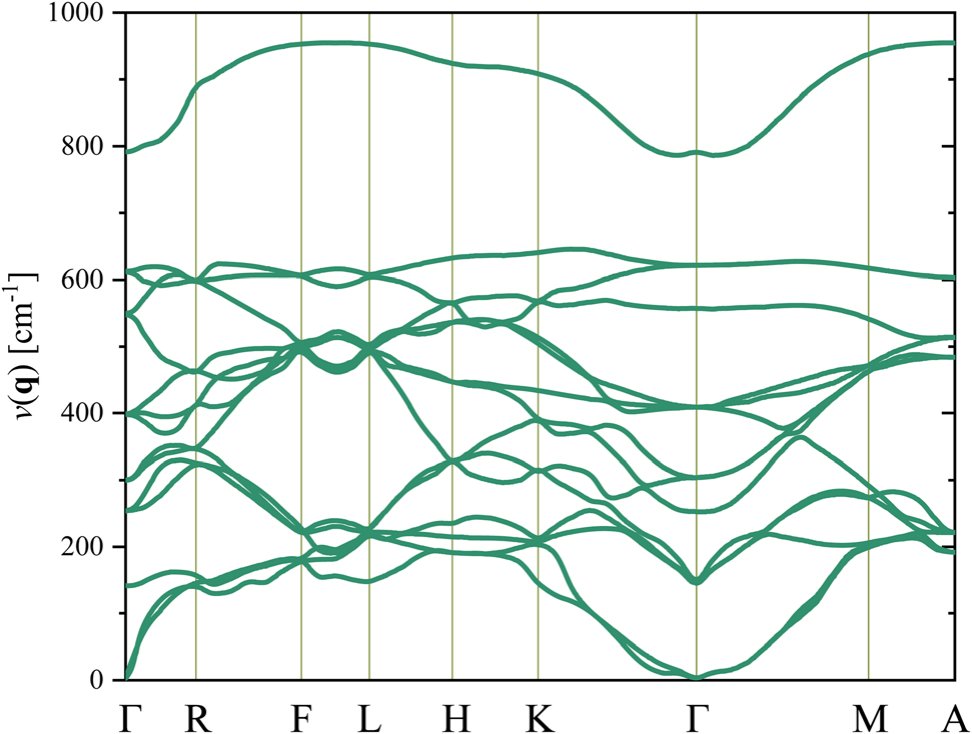
The phonon spectrum of the *Cm*(M_B_) phase Pb(Zr_0.5_Ti_0.5_)O_3_. The abscissa is the high symmetry point located in the first Brillouin zone (Γ, R, F, L, H, K, M, and A). Their local coordinates are (0, 0, 0), (0, *ζ*, 0), (*ζ*, *ζ*, 0), (*ζ*, 0, 0), (*ζ*, *ζ*, *ζ*), (0, 0, *ζ*), (0, *ζ*, *ζ*), (*ζ*, 0, *ζ*), respectively.

In Fig. 3, firstly, it is found that the degeneracy of the phonon structure of the *Cm*(M_B_) phase is destroyed compared with the literature. The first reason for this result is that the *Cm*(M_B_) phase belongs to the lower crystal group, and the symmetry of the structure is reduced. Secondly, it is due to the doping effect of Zr atoms in the crystal. The softest frequency is 2.72 cm^−1^, and the second softest frequency is 2.9 cm^−1^. They are all located at *Γ* point, which belongs to a vibration of the optical transverse mode of the central atom of the lattice. Finally, since the lowest frequency vibration mode of *Cm*(M_B_) phase is above the virtual frequency region, it can be considered that *Cm*(M_B_) phase can exist stably in theory.

This stability of the monoclinic structure is reflected in its ferroelectric property. For the *Cm*(M_B_) phase located at *x* = 0.5, the component of the polarization intensity vector is *P*_*x*_ = 0.133, *P*_*y*_ = 0.125, and *P*_*z*_ = 0.0457, respectively. This indicates that *Cm*(M_B_) phase may have different displacement polarization characteristics and polarization rotation characteristics from other structures. This phenomenon should first be reflected in the chemical bond changes in the structure. For PZT, the covalent bond between A position atoms and O with the covalent bond between the central atoms and O2p orbit are equally important contributions to the stability of structure.^51–53^ The Born effective charge in the range of 0.45 < *x* < 0.55 and the displacement of the atom with respect to the symmetric position are shown in Table S2.† The Z* stands for the average Born effective charge, and the 
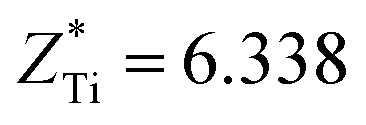
, 
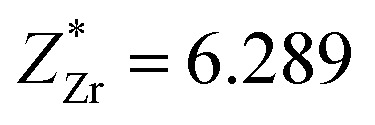
. These two very close values indicate that the two atoms have very close chemical environments.^47^ Additionally, both 
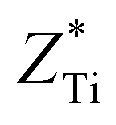
 and 
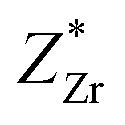
 are greater than its valency of +4; this suggests that the chemical bond between Ti, Zr, and O is mainly covalent (*Z*_O1*x*_ = −2.494, *Z*_O1*y*_ = −4.429, *Z*_O1*z*_ = −2.209, *Z*_O3*x*_ = −2.381, *Z*_O3*y*_ = −2.341, *Z*_O3*z*_ = −4.684, *Z*_O4*x*_ = −2.75, *Z*_O4*y*_ = −4.846, *Z*_O4*z*_ = −1.966, *Z*_O6*x*_ = −2.106, *Z*_O6*y*_ = −2.041, *Z*_O6*z*_ = −4.058). Meanwhile, the 
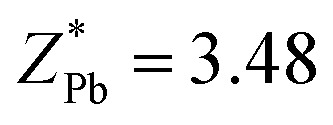
, which may indicate the weakening of the covalent bond between Pb6s and O2p orbit compared with the *P*4*mm* structure in this chemical compound. Finally, the most stable structure around *x* = 0.5 is the *R*3̄*m* phase,^54^ which has a similar band gap to the *Cm*(M_B_) structure shown in Fig. S5.† This suggests that the *Cm*(M_B_) structure may have an electronic structure with a reduced DOS of conduction bond and an increased DOS of valence band.

**Fig. 4:**
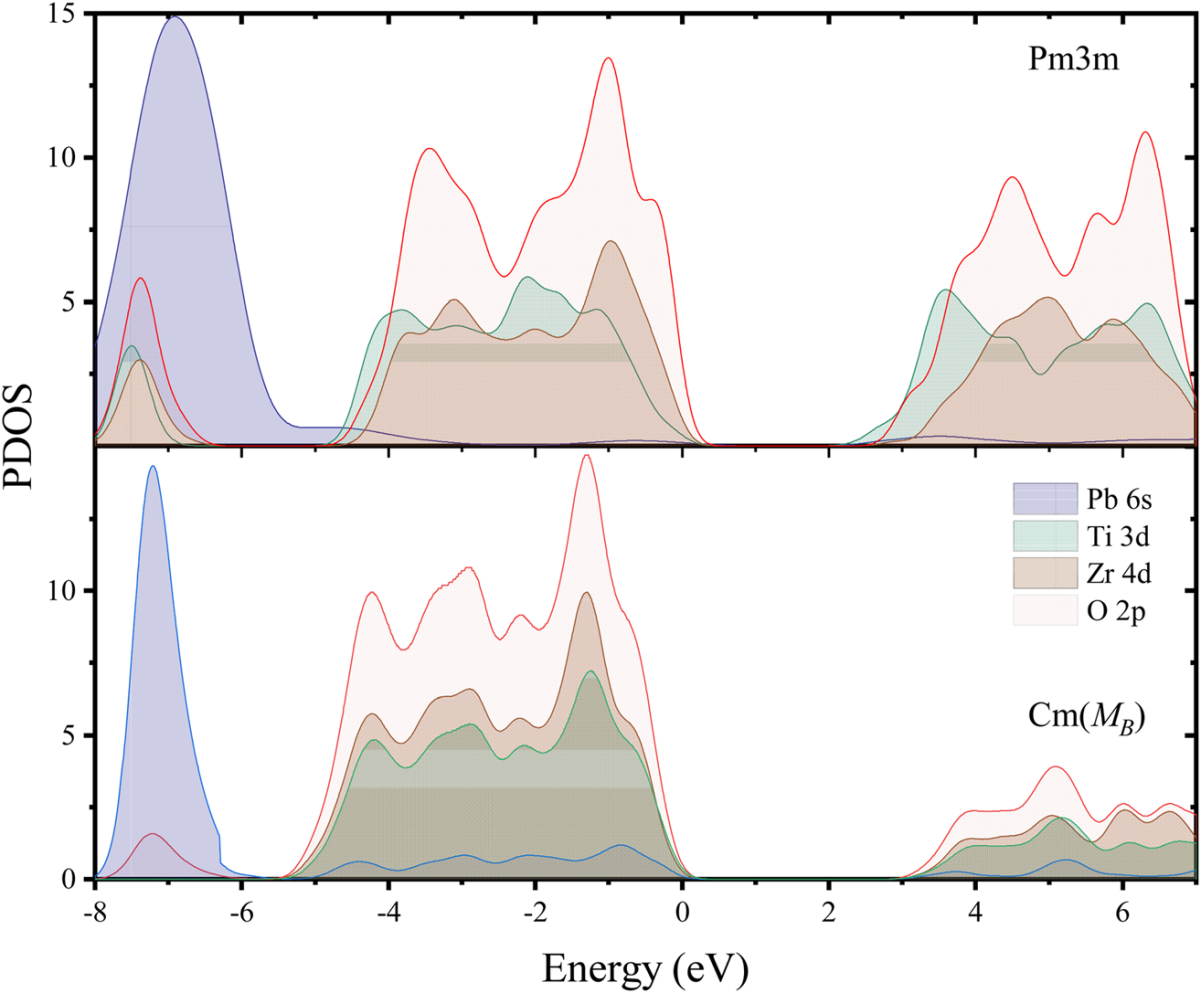
The density of states (DOS) for the *Pm*3*m* phase (above) and *Cm*(M_B_) (bottom) phases located at *x* = 0.5.

Fig. 4 shows the structure of covalent bonds by calculating the PDOS method. It can be observed firstly that both phases exhibit large band gaps, with *E*_g*Cm*(M_B_)_ = 2.987 eV, and *E*_g*Pm*3*m*_ = 2.463 eV, respectively. Fig. S7† shows the electronic structure of the M_B_-*Cm* phase. Additionally, the PDOS spectra of the two phases exhibit similar peak shapes and identical positions. The peak position of the *Cm*(M_B_) phase is closer to lower energy (*Cm*(M_B_): −5.5 eV to 0 eV, *Pm*3*m*: −5 eV to 0 eV) indeed due to the reduction of the total DOS visible in the conduction band. Fig. S8† provides some electronic structures of PZT in different phases. Unlike phase transitions in general materials, the ferroelectric phase transition in the PZT cells is entirely determined by the distortion of oxygen octahedra in the lattice. As can be seen in Fig. S8,† the band structures and DOS between different phases are very similar. Therefore, despite the inherent lack of rigor in this approach, it is possible to determine band shifts by assessing the band gaps of these structures. This difference is mainly the contribution of Ti and Zr atoms in the valence band. This can be attributed to atomic displacements caused by the phase transition process. In PDOS, the range from −5 eV to 0 eV primarily consists of hybridization peaks between the oxygen and the central atoms. The main contributors to the p-orbit DOS are O2p orbit, while the main contributors to the d-orbit DOS are Ti3d and Zr4d orbits. Above the Fermi level, the O2p with Ti3d and Zr4d orbits exhibit similar peak shapes and peak positions. Meanwhile, even though in the conduction band, *Cm*(M_B_) has lower DOS than *Pm*3*m* phase, *Cm*(M_B_) crystal still occupies higher energy positions (*Cm*(M_B_): 3 eV, *Pm*3*m*: 2 eV); this effect plays a significant role in the stability of the *Cm*(M_B_) phase. This indicates a weak hybridization between the O2p and d orbits of central atoms, suggesting a weakening bonding energy in the Ti–O and Zr–O bonds. At −8 eV to −6 eV, the DOS of Pb6s orbit is drastically reduced compared to *Pm*3*m*. Table S3† shows the –COHP of the different PZT phases. Due to the limitations of the VCA, these calculations are based on 2 × 4 × 4 supercells, where the atomic distribution belongs to the *T*_d_ point group, representing a crystal with a Zr/Ti ratio of 1. In the *Pm*3*m* phase, the –COHP values for Zr–O and Ti–O are 0.0431 and 0.0427, respectively. Similarly, for commonly used ferroelectric structures such as *P*4*mm* and *R*3*m*, the –COHP values also show a decrease. In the *Cm*(M_B_) structure, the –COHP is the lowest, with values of 0.0406 and 0.0395 for Ti–O_1_ and Ti–O_2_, respectively, and 0.0412 and 0.0409 for Zr–O_1_ and Zr–O_2_, respectively. This indicates a reduction in the overlap of bonding orbits between the central atoms and the oxygen atoms in the *Cm*(M_B_) structure, which is consistent with the conclusion of weakened covalent bonds obtained from the PDOS analysis.

The above conclusions based on PDOS are consistent with the conclusions drawn by the study of Born effective charge in the previous paragraphs of the paper. In the electron density image presented in Fig. S2† the monoclinic structure was selected as the study object. In the 2 × 2 × 2 VCA supercell constructed in this paper, all parameters are fully relaxed. By examining the electron density images of the central atom plane and the O3, O4 atom plane in the (001) direction of the crystal, the charge density transfer between the central atom and the O3, O4 atoms are found. This effect may relate to the contraction or expansion of the oxygen octahedron structure in this direction. In addition, the antiferroelectric distortion belonging to the M_2_^+^ mode can be observed in the fully relaxed supercell.^55–58^ This is preliminarily believed to be related to the monoclinic structure reducing its own energy and improving the stability.

Based on the calculated ferroelectric and elastic properties of the crystal, the piezoelectric constants can be obtained from the rate of change of polarization intensity with respect to strain,^49,59,60^ where the strain is set to not exceed 1%. Firstly, when the external electric field is zero, the piezoelectric stress constants can be expressed as:4
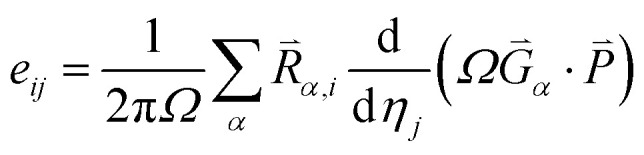
In practice, this relationship is usually expressed in two parts:5
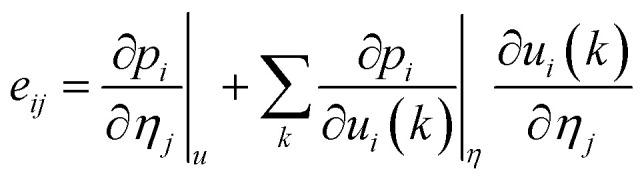
In these equations, *Ω* represents the unit cell volume, *R* and *G* denote real-space and reciprocal-space vectors, respectively, *η* stands for macroscopic strain, and the summation range *α* extends over three dimensions, *u* is the intracell strain or called the intrinsic parameter, and the range of *k* includes all the atoms. Under these conditions, polarization is contributed by the following two components:

6 *p*_*i*_ = *p*^s^_*i*_ + *e*_*ij*_*η*_*j*_

where *p*^s^ represents the material's spontaneous polarization. Thus, for a monoclinic structure material, the change in polarization along the three directions can be expressed as follows:7
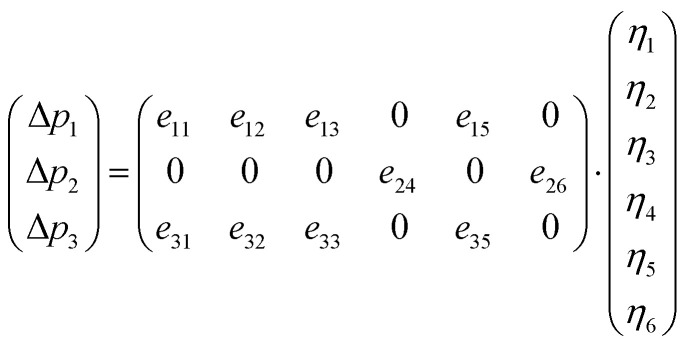


For the monoclinic structure in *m* space group, the elastic constant matrix is:8
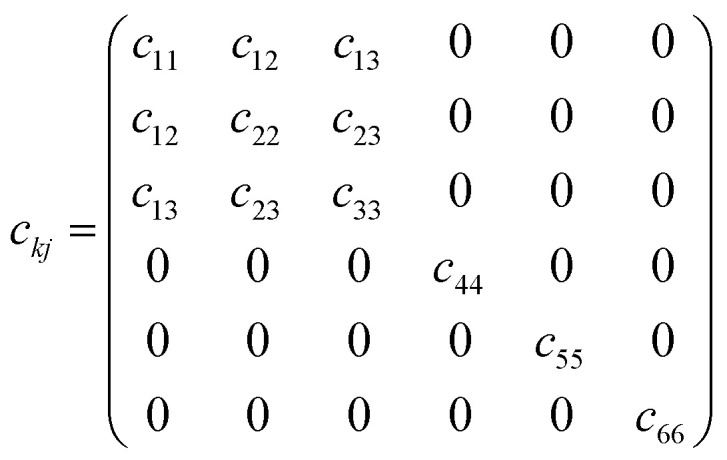


The relationship between them is as follows:

9 *e*_*ij*_ = *d*_*ik*_*c*_*kj*_

This relationship can be reduced to:10
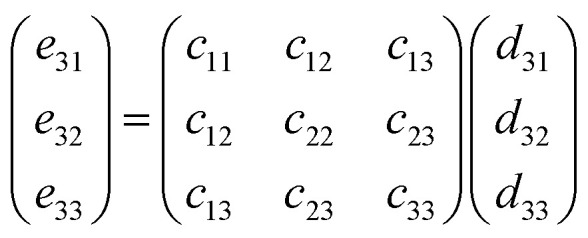


Taking the polarization intensity along the [001] direction as an example, it can be expressed as:

11 Δ*p*_3_ = *e*_31_*η*_1_ + *e*_32_*η*_2_ + *e*_33_*η*_3_ + *e*_35_*η*_5_

By applying strain along the *c*-axis, the piezoelectric constant can be obtained as:12
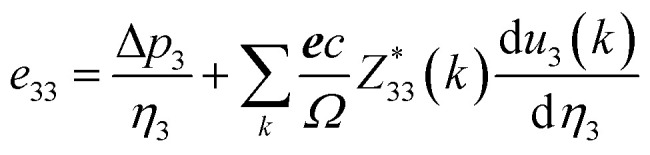


The piezoelectric strain constant *d*_*ij*_ is related to this formula. The bold ***e*** is the meta charge. *Z* means Born effective charge.

13 *d*_33_ ≈ *e*_33_*s*_33_

where *s*_*ij*_ represents the calculated elastic compliance constant.

**Fig. 5:**
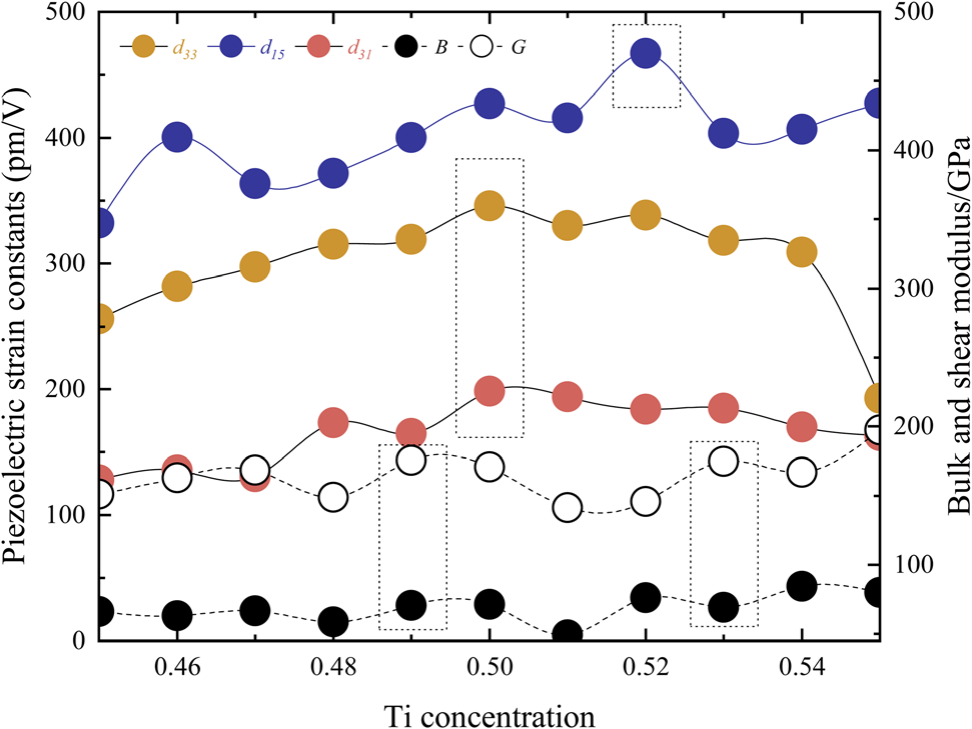
The piezoelectric strain constants and elastic properties in the *Cm*(M_B_) phase.

Fig. 5 presents the relationship between piezoelectric strain constants and Ti concentration. The maximum value of the *d*_33_ = 345.71 pm V^−1^ is observed at *x* = 0.5. The maximum value of the *d*_15_ = 466.84 pm V^−1^ is found at *x* = 0.52. Similarly, the maximum value of the *d*_31_ = −198.33 pm V^−1^ is at *x* = 0.5. These positions are in close proximity to MPB. Compared to experimental values, the *Cm*(M_B_) phase exhibits lower values.^1,2^ However, although the highest performance of the structure is a shear piezoelectric coefficient, the relatively high *d*_31_ in the *Cm*(M_B_) phase suggests that the structure possesses distinctive transverse piezoelectricity. This is because the above three extreme values are not located in the same chemical composition. Due to the differing symmetries of various ferroelectric structures, utilizing a single metric such as the *c*/*a* ratio to measure lattice distortion is not universally applicable. In Fig. S9,† the volume and lattice parameters decrease with increasing *x*, while the displacement of the central atom relative to the crystal center remains essentially unchanged. This indicates that as *x* increases, the lattice distortion caused by the central atom intensifies, which is consistent with the behavior of PZT near MPB.

A comparison of the piezoelectric properties of several monoclinic structures at the MPB is presented in Table 1. Among these monoclinic structures, the M_B_ structure exhibits the highest piezoelectric performance; however, this performance still shows a significant gap compared to the rhombohedral and tetragonal structures. For instance, the most successful commercial PZT exhibits a *d*_33_ of 750 pm V^−1^ at the MPB, while the common *P*4*mm* and *R*3̄*m* phases of PZT have *d*_33_ values of 260 pm V^−1^ and 225 pm V^−1^, respectively. By exhaustively exploring all possible structures at the MPB based on the VCA method, we demonstrate that the intrinsic piezoelectricity of monoclinic structures is not the main contributor to the MPB effect.

**Table 1:** Comparison of the piezoelectric properties of each structure at the MPB

pm V^−1^	*P*4*mm*	*R*3̄*m*	*R*3̄*c*	*Cm*(M_A_)	*Cm*(M_B_)	*Pm*(M_C_)	*Cc*
*d*_33_	454.76	473.46	442.33	281.17	345.71	212.36	171.34
*d*_15_	583.67	615.14	501.82	301.12	466.84	251.37	204.71

Fig. S3† illustrates the total energy, the zero point energy, and the frequency of the softest phonon vibration mode at *Γ* point of the structure around *x* = 0.5. It is shown that *x* = 0.5 and *x* = 0.52 are both located at the bottom of the energy curve, while the softest frequency of *x* = 0.5 is lower and reaches the range of virtual frequencies. Fig. S4† shows the above properties for *x* = 0.52 *versus c*/*a*. These data suggest that the structure with *x* = 0.52 is more stable indeed, while the high piezoelectric coefficient *d*_33_ and *d*_31_ at *x* = 0.5 may be related to the smaller virtual frequency here, due to the softening of the transverse mode vibration of the central atoms. Fig. 5 also depicts the bulk modulus *B* and shear modulus *G*. These parameters are derived from the elastic stiffness constants *c*_*ij*_ and the elastic compliance constants *s*_*ij*_. The computed results show the maximum and minimum values at *x* = 0.49, *x* = 0.53, and *x* = 0.51, respectively, for *B* and *G*. These extrema positions do not align with the position in which it has the direction of maximum polarization, indicating that such elastic properties may play a significant role in influencing the piezoelectric performance. Additionally, these extreme values stand vary close (*s*_31,*x*=0.5_ = 31.28, *s*_31,*x*=0.52_ = 29.53, *s*_33,*x*=0.5_ = 34.86, *s*_33,*x*=0.52_ = 34.24, *s*_15,*x*=0.5_ = 22.02, *s*_15,*x*=0.52_ = 17.99, unit: 10^−12^ m^2^ n^−1^). The difference in piezoelectric properties is almost due to differences in electronic structure (*e*_31,*x*=0.5_ = −6.34, *e*_31,*x*=0.52_ = −6.23, *e*_33,*x*=0.5_ = 9.92, *e*_33,*x*=0.52_ = 9.88, *e*_15,*x*=0.5_ = 19.41, *e*_15,*x*=0.52_ = 25.94, unit: C m^2^). Based on this conclusion, the polarization rotation theory is adopted in this paper. By applying strain to the model, the position of the central atoms is changed, and the rotation of the polarization direction of the structure to the *d* position is simulated.

**Fig. 6:**
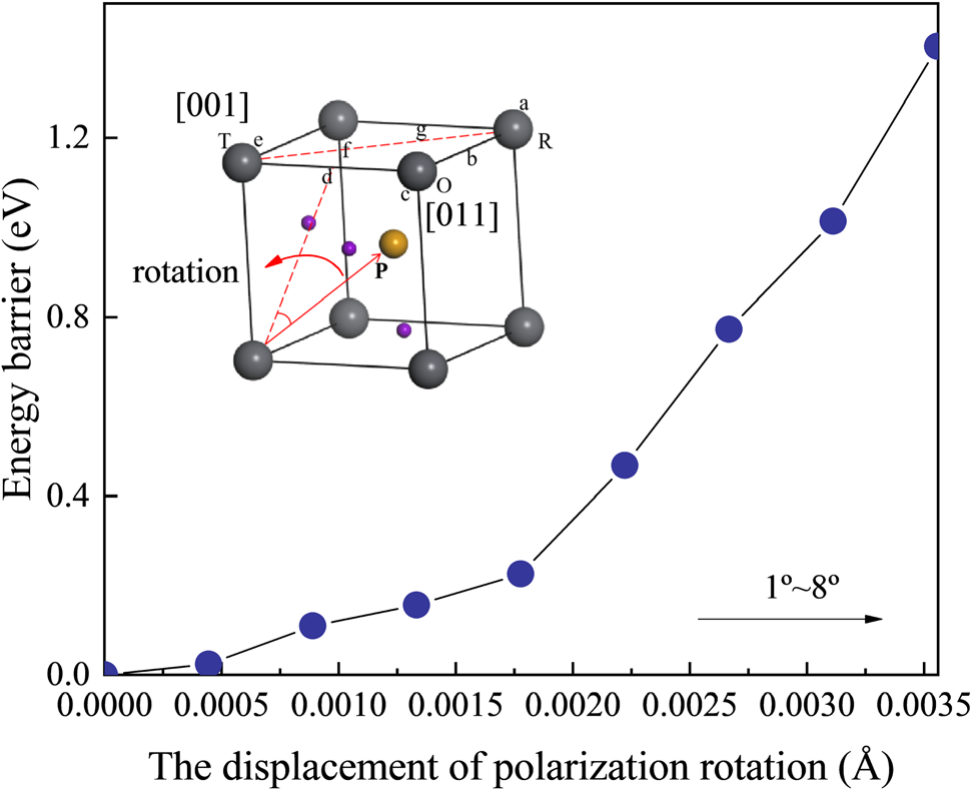
Portrayal of the energy barriers along the polarization rotation path of *Cm*(M_B_) phase along the *d* direction at *x* = 0.52.

Fig. S6† illustrates the total energy, the zero point energy, and the frequency of the softest phonon vibration mode at *Γ* point of the structure located at *x* = 0.52. The energy of barriers along the polarization rotation path is defined *via* the formula *E*_B_(*θ*) = *E*_total energy_(*θ*) − *E*_total energy_(0). *θ* is the angle of polarization rotation. It can be observed from Fig. 6 that the barrier height gradually increases with the increase in the rotation angle. Moreover, within the lower rotation angle, relatively low barrier heights are observed, corresponding to the flattening of free energy. This phenomenon is also found in the zero point energy of Fig. S6.† This serves as the origin of the intrinsic piezoelectricity in the *Cm*(M_B_) phase.

## 4. Conclusion

In this study, the intrinsic piezoelectric properties of monoclinic PZT were investigated *via* first-principles study. Firstly, crystal structures *versus* Ti concentrations were constructed based on VCA, and the position of the MPB was determined at *x* ∼ 0.53. The free energy indicates the relatively lower of *Cm*(M_B_) phase at 0.475 < *x* < 0.512. The study of the phonon spectrum shows that the structure has the softest mode indicating stable existence. Secondly, studies on the electronic structure and Born effective charge show that this stability is inseparable from the bond energy enhancement between the O and central atoms. Subsequently, the elastic and piezoelectric properties of the crystal were calculated. The results reveal relatively lower values of the *d*_33_ and *d*_15_ piezoelectric strain constants in the *Cm*(M_B_) phase, being 345.71 pm V^−1^ (*x* = 0.5) and 466.84 pm V^−1^ (*x* = 0.52), respectively. However, the *d*_31_ component of the piezoelectric strain constant was found to be relatively high, at −198.33 pm V^−1^ (*x* = 0.5). By exhaustively exploring all possible structures at the MPB, we demonstrate that the intrinsic piezoelectricity of monoclinic structures is not the main contributor to the MPB effect. Finally, the origin of this piezoelectricity was explained by calculating the energy barriers along the polarization rotation path. Due to the flattening of the free energy, the *Cm*(M_B_) phase of PZT crystals exhibits relatively high intrinsic piezoelectricity. This may bring new ideas to the study of the MPB effect.

## Data availability

All authors in this paper promise that all data do not exist in any published articles and all data are free from any restrictions involving privacy and secrecy.

## Conflicts of interest

There are no conflicts to declare.

## Supplementary Material

RA-014-D4RA06270A-s001
