# Supplementary material for: The intrinsic piezoelectricity of monoclinic Pb(Zr1−xTix)O3
Source: RSC Adv. 2024 Dec 2;14(51):38245–52. doi: 10.1039/d4ra06270a (PMC11610901; doi:10.1039/d4ra06270a)
Supplement: RA-014-D4RA06270A-s001 [file RA-014-D4RA06270A-s001.pdf]

Supplementary Information (SI) for RSC Advances.
This journal is © The Royal Society of Chemistry 2024

Theoretical calculation of intrinsic piezoelectricity of monoclinic lead zirconate titanate

Junyu Niu [a],Chong Li [b],Zengzhe Xi[a, *]
[a] Shaanxi Key Laboratory of Photoelectric Functional Materials and Devices, School of Materials and Chemical Engineering, Xi'an Technological University, Xi'an 710021, Shaanxi, China
[b] Shenzhen Key Laboratory of Advanced Thin Films and Applications, Key Laboratory of Optoelectronic Devices and Systems of Ministry of Education and Guangdong Province, College of Physics and Optoelectronic Engineering, Shenzhen University, Shenzhen 518060, P. R. China
[*] Corresponding author, E-mail: zzhxi@xatu.edu.cn

Supplemental Material

TableS1. Summarization of the lattice parameters of PZT models.

space group	ti concentration	a/Å	b/Å	c/Å	alpha/(º)	beta/(º)	gamma/(º)	volume/Å^3
r3m	0	4.174	4.174	4.174	89.65	90.00	90.00	72.583
r3m	0.1	4.149	4.149	4.149	89.61	90.00	90.00	71.263
r3m	0.2	4.117	4.117	4.117	89.56	90.00	90.00	69.654
r3m	0.3	4.089	4.089	4.089	89.52	90.00	90.00	68.219
r3m	0.4	4.063	4.063	4.063	89.47	90.00	90.00	66.943
r3m	0.5	4.039	4.039	4.039	89.43	90.00	90.00	65.774
r3m	0.6	4.018	4.018	4.018	89.38	90.00	90.00	64.755
r3m	0.7	3.999	3.999	3.999	89.34	90.00	90.00	63.815
r3m	0.8	3.975	3.975	3.975	89.29	90.00	90.00	62.678
p4mm	0	4.123	4.123	4.245	90.00	90.00	90.00	72.162
p4mm	0.1	4.102	4.102	4.200	90.00	90.00	90.00	70.673
p4mm	0.2	4.084	4.084	4.159	90.00	90.00	90.00	69.380
p4mm	0.3	4.063	4.063	4.122	90.00	90.00	90.00	68.051
p4mm	0.4	4.041	4.041	4.095	90.00	90.00	90.00	66.859
p4mm	0.5	4.018	4.018	4.069	90.00	90.00	90.00	65.707
p4mm	0.6	3.980	3.980	4.047	90.00	90.00	90.00	64.103
p4mm	0.7	3.976	3.976	4.032	90.00	90.00	90.00	63.738
p4mm	0.8	3.956	3.956	4.032	90.00	90.00	90.00	63.097
p4mm	0.9	3.930	3.930	4.056	90.00	90.00	90.00	62.640
p4mm	1	3.898	3.898	4.125	90.00	90.00	90.00	62.677
mb-cm	0.2	4.123	4.123	4.115	90.35	90.00	89.74	69.951
mb-cm	0.3	4.098	4.098	4.106	90.40	90.00	89.69	68.956
mb-cm	0.4	4.077	4.077	4.085	90.44	90.00	89.65	67.901
mb-cm	0.5	4.057	4.057	4.049	90.49	90.00	89.60	66.644

mb-cm	0.6	4.038	4.038	4.050	90.53	90.00	89.56	66.037
mb-cm	0.7	4.021	4.021	4.033	90.58	90.00	89.51	65.207
r3c	0	4.152	4.152	5.085	90.00	90.00	90.00	87.479
r3c	0.1	4.123	4.123	5.051	90.00	90.00	90.00	85.692
r3c	0.2	4.098	4.098	5.019	90.00	90.00	90.00	84.113
r3c	0.3	4.072	4.072	4.988	90.00	90.00	90.00	82.555
r3c	0.4	4.051	4.051	4.962	90.00	90.00	90.00	81.286
r3c	0.5	4.027	4.027	4.933	90.00	90.00	90.00	79.852
r3c	0.6	4.008	4.008	4.909	90.00	90.00	90.00	78.696
r3c	0.7	3.988	3.988	4.885	90.00	90.00	90.00	77.553
r3c	0.8	3.975	3.975	4.869	90.00	90.00	90.00	76.773
mc-pm	0.2	4.019	3.852	4.134	90.00	90.15	90.00	63.872
mc-pm	0.3	4.015	3.892	4.168	90.00	90.20	90.00	65.006
mc-pm	0.4	4.011	3.932	4.207	90.00	90.24	90.00	66.225
mc-pm	0.5	4.007	3.972	4.250	90.00	90.28	90.00	67.512
mc-pm	0.6	4.003	4.009	4.281	90.00	90.31	90.00	68.550
pbam	0	5.878	11.79	8.192	90.00	90.00	90.00	567.781
pm3m	0	3.973	3.973	3.973	90.00	90.00	90.00	62.732
pm3m	0.1	3.996	3.996	3.996	90.00	90.00	90.00	63.830
pm3m	0.2	4.020	4.020	4.020	90.00	90.00	90.00	64.947
pm3m	0.3	4.043	4.043	4.043	90.00	90.00	90.00	66.083
pm3m	0.4	4.066	4.066	4.066	90.00	90.00	90.00	67.240
pm3m	0.5	4.090	4.090	4.090	90.00	90.00	90.00	68.417
pm3m	0.6	4.114	4.114	4.114	90.00	90.00	90.00	69.614
pm3m	0.7	4.138	4.138	4.138	90.00	90.00	90.00	70.832
pm3m	0.8	4.162	4.162	4.162	90.00	90.00	90.00	72.072
pm3m	0.9	4.186	4.186	4.186	90.00	90.00	90.00	73.333
pm3m	1	4.210	4.210	4.210	90.00	90.00	90.00	74.617
ma-cm	0.2	4.168	4.168	4.166	90.27	90.00	89.87	72.235
ma-cm	0.3	4.125	4.125	4.123	90.31	90.00	89.82	70.000
ma-cm	0.4	4.098	4.098	4.096	90.36	90.00	89.78	68.636
ma-cm	0.5	4.078	4.078	4.076	90.40	90.00	89.73	67.662
ma-cm	0.6	4.057	4.057	4.055	90.45	90.00	89.69	66.623
ma-cm	0.7	4.050	4.050	4.048	90.49	90.00	89.65	66.255
p1	0.5	4.102	4.095	4.412	89.31	89.97	90.00	73.958

$Pb(Zr_{1-x}Ti_x)O_3$ crystals is Pm3m phase above the *Tc*, and exhibit different spontaneous polarization directions, such as P4mm, R3-m, R3-c, and Cm phases etc, below *Tc* as Ti concentration changes. We employed VCA to vary the Ti and Zr atom ratio of these models continuously and remove the parameters of structures which fail to converge. The BFGS algorithm was employed to perform geometry optimization for these crystal structures.

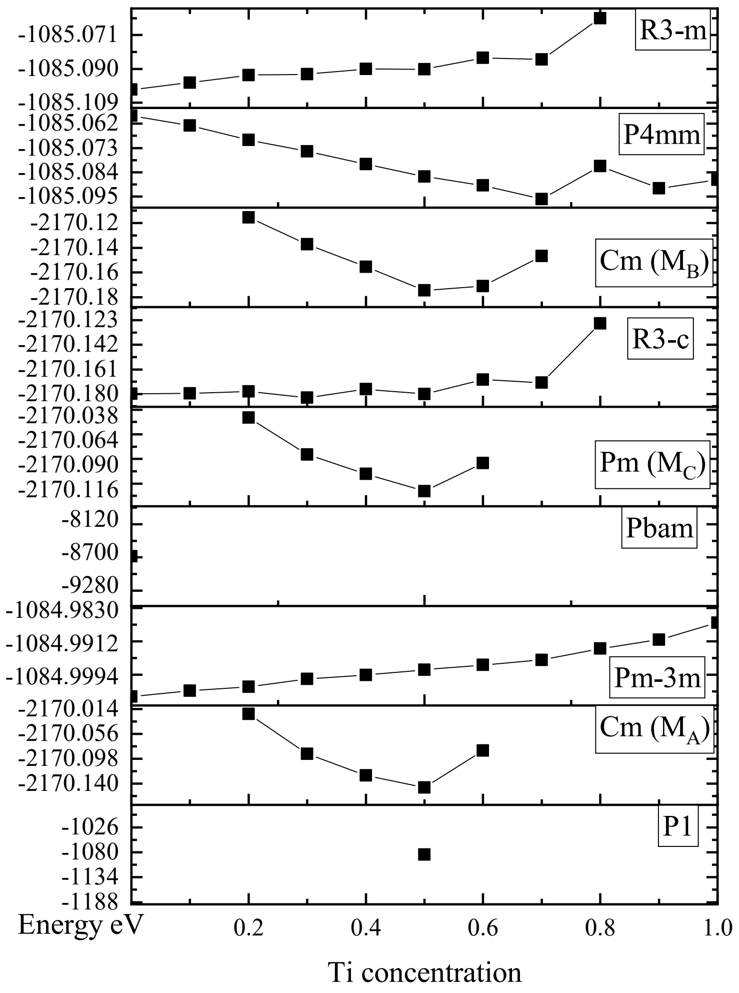

FigS1. The total energy of PZT models.

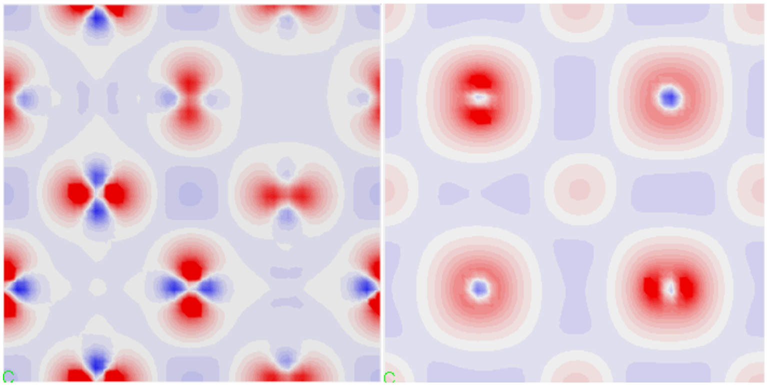

FigS2. The charge density distribution of monoclinic PZT models.

The charge density distribution along the direction [001], the upper left corner is the direction [011], and the lower left corner is the direction [111]. The model is constructed based on the VCA, x=0.52 monoclinic PZT supercell, the former is near the O_1 atom plane, the latter is the central atom plane, after sufficient structural relaxation, the model shows a certain degree of M_2^+ antiferroelectric distortion.

TableS2. The displacement and Born effective charge of monoclinic PZT models.

Ti concentration	0.45	0.46	0.47	0.48	0.49	0.5	0.51	0.52	0.53	0.54	0.55

pb1x	0	0	0	0	0	0	0	0	0	0	0
pb1y	0	0	0	0	0	0	0	0	0	0	0
pb1z	0	0	0	0	0	0	0	0	0	0	0
pb2x	0.001	0.002	0.003	0.003	0.003	0.003	0.004	0.003	0.002	0.002	0.002
pb2y	0.002	0.002	0.003	0.002	0.002	0.002	0.002	0.003	0.004	0.003	0.002
pb2z	0.002	0.002	0.002	0.001	0.001	0.002	0.002	0.002	0.002	0.002	0.001
tix	0.041	0.042	0.043	0.043	0.044	0.043	0.042	0.042	0.043	0.043	0.044
tiy	0.041	0.042	0.042	0.041	0.040	0.041	0.042	0.042	0.042	0.042	0.042
tiz	0.016	0.016	0.016	0.016	0.016	0.016	0.016	0.015	0.015	0.015	0.015
zrx	0.067	0.070	0.073	0.072	0.071	0.071	0.070	0.071	0.073	0.069	0.067
zry	0.069	0.069	0.070	0.068	0.067	0.068	0.070	0.068	0.067	0.068	0.069
zrz	0.008	0.009	0.009	0.009	0.009	0.009	0.008	0.009	0.009	0.009	0.009
o1x	0.059	0.058	0.056	0.057	0.059	0.059	0.057	0.059	0.062	0.059	0.057
o1y	0.042	0.043	0.044	0.041	0.037	0.040	0.042	0.042	0.042	0.044	0.046
o1z	0.013	0.013	0.013	0.013	0.013	0.013	0.014	0.013	0.013	0.013	0.014
o3x	0.056	0.062	0.066	0.067	0.066	0.064	0.061	0.064	0.067	0.066	0.067
o3y	0.063	0.063	0.064	0.061	0.058	0.059	0.061	0.060	0.059	0.058	0.057
o3z	0.011	0.011	0.010	0.010	0.010	0.010	0.011	0.011	0.012	0.011	0.011
o4x	0.048	0.048	0.049	0.049	0.048	0.051	0.053	0.051	0.048	0.052	0.057
o4y	0.002	0.002	0.002	0.002	0.002	0.002	0.002	0.002	0.002	0.002	0.002
o4z	0.011	0.011	0.012	0.011	0.011	0.011	0.011	0.012	0.012	0.011	0.011
o6x	0.051	0.055	0.061	0.056	0.053	0.054	0.055	0.057	0.060	0.057	0.054
o6y	0.055	0.058	0.060	0.058	0.057	0.056	0.055	0.055	0.056	0.054	0.051
o6z	0.010	0.010	0.010	0.011	0.011	0.011	0.011	0.010	0.010	0.010	0.010
zpb1x	3.431	3.645	3.808	3.804	3.845	3.815	3.770	3.702	3.695	3.598	3.431
zpb1y	3.619	3.646	3.732	3.567	3.431	3.622	3.770	3.747	3.657	3.579	3.544
zpb1z	3.290	3.363	3.395	3.266	3.150	3.318	3.500	3.469	3.465	3.455	3.465
zpb2x	3.871	3.808	3.715	3.677	3.715	3.839	3.910	3.778	3.675	3.745	3.793
zpb2y	3.793	3.670	3.519	3.491	3.519	3.700	3.910	3.855	3.793	3.739	3.715
zpb2z	2.646	2.616	2.592	2.538	2.484	2.589	2.700	2.640	2.565	2.502	2.430
ztix	6.442	6.514	6.509	6.510	6.576	6.583	6.710	6.449	6.240	6.328	6.442
ztiy	6.555	6.489	6.295	6.386	6.425	6.406	6.490	6.380	6.360	6.135	5.971
ztiz	6.166	6.068	5.922	5.923	6.044	6.026	6.105	6.092	5.983	5.941	5.983
zzrx	5.656	5.627	5.656	5.828	5.966	6.133	6.215	6.110	6.029	6.227	6.339
zzry	6.029	5.897	5.718	5.960	6.215	6.215	6.215	6.103	6.029	5.912	5.842
zzrz	6.183	6.140	6.183	6.229	6.251	6.518	6.721	6.802	6.788	6.802	6.788
zo1x	-2.652	-2.587	-2.548	-2.483	-2.418	-2.494	-2.600	-2.517	-2.418	-2.459	-2.496
zo1y	-4.078	-4.125	-4.256	-4.357	-4.433	-4.429	-4.433	-4.411	-4.389	-4.428	-4.433
zo1z	-2.020	-2.025	-1.998	-2.098	-2.220	-2.209	-2.220	-2.202	-2.176	-2.187	-2.220
zo3x	-2.309	-2.410	-2.479	-2.425	-2.333	-2.381	-2.430	-2.328	-2.211	-2.272	-2.333
zo3y	-2.309	-2.321	-2.357	-2.321	-2.284	-2.341	-2.430	-2.344	-2.211	-2.304	-2.406
zo3z	4.365	4.455	4.608	4.526	4.462	4.684	4.850	4.767	4.608	4.594	4.608
zo4x	-2.805	-2.745	-2.695	-2.720	-2.723	-2.750	-2.750	-2.665	-2.613	-2.582	-2.558
zo4y	-4.548	-4.656	-4.792	-4.749	-4.792	-4.846	-4.890	-4.851	-4.792	-4.632	-4.499

zo4z	-1.950	-1.918	-1.891	-1.901	-1.950	-1.966	-1.990	-1.954	-1.910	-1.915	-1.950
zo6x	-2.142	-2.131	-2.142	-2.113	-2.100	-2.106	-2.100	-2.111	-2.121	-2.108	-2.058
zo6y	-2.121	-2.016	-1.911	-1.933	-1.995	-2.041	-2.100	-1.985	-1.911	-1.939	-1.932
zo6z	-3.860	-3.974	-4.026	-3.988	-3.943	-4.058	-4.150	-4.142	-4.109	-4.083	-4.026

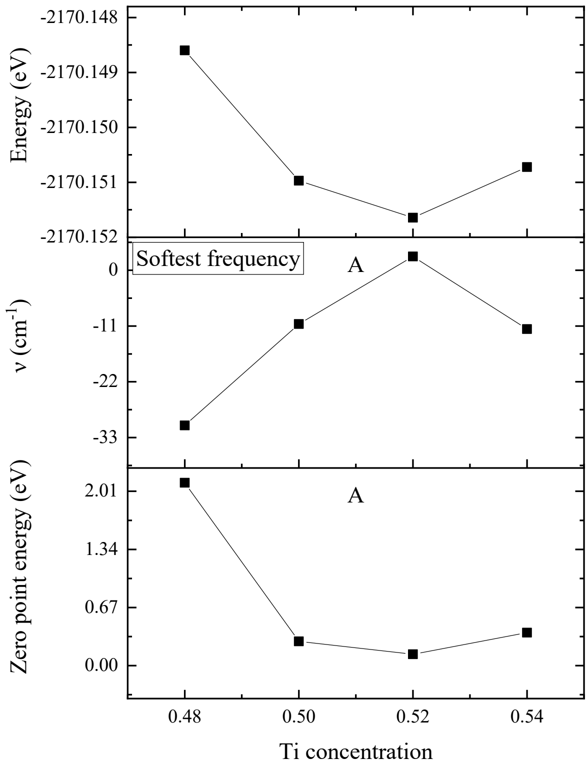

FigS3. The total energy, softest frequency, and zero point energy versus Ti concentration near x=0.5 of monoclinic PZT.

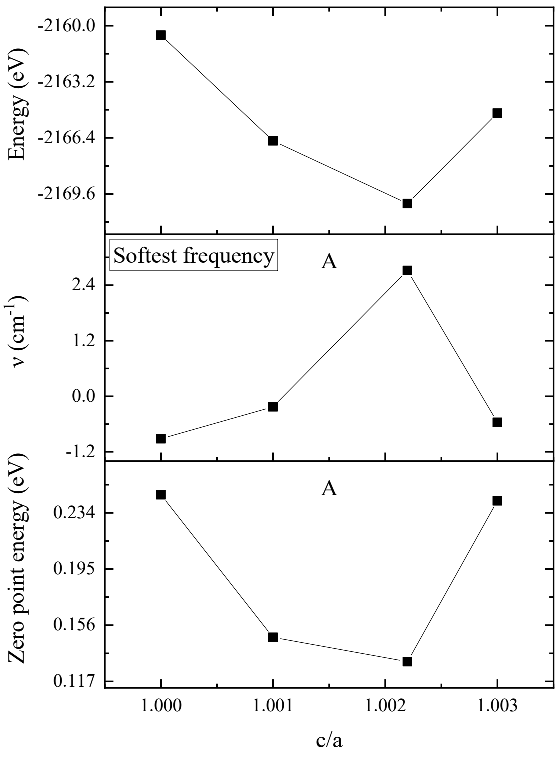

FigS4. The total energy, softest frequency, and zero point energy versus lattice parameters ratio c/a of x=0.52.

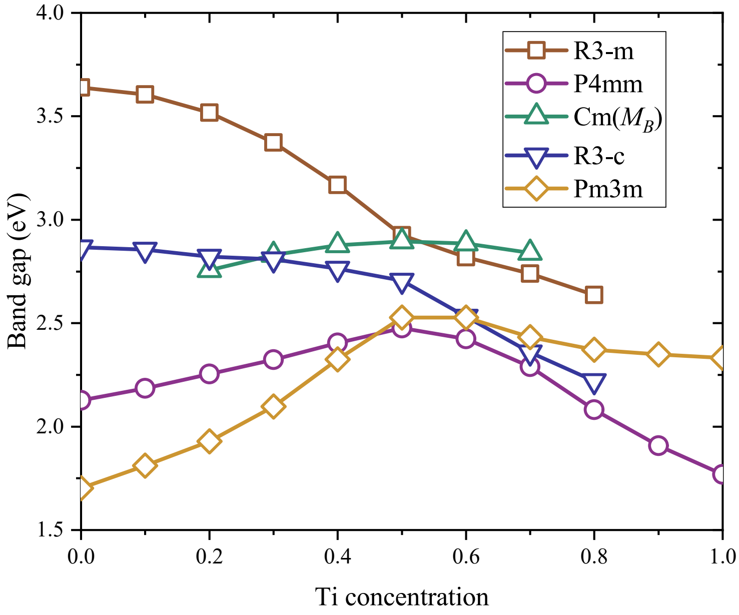

FigS5. The band gap of PZT models.

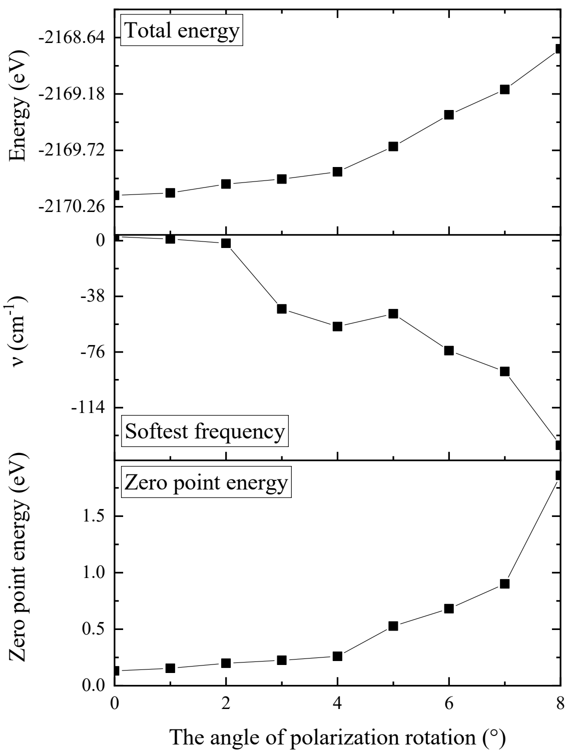

FigS6. The total energy, softest frequency, and zero point energy versus the angle of polarization rotation of x=0.52.

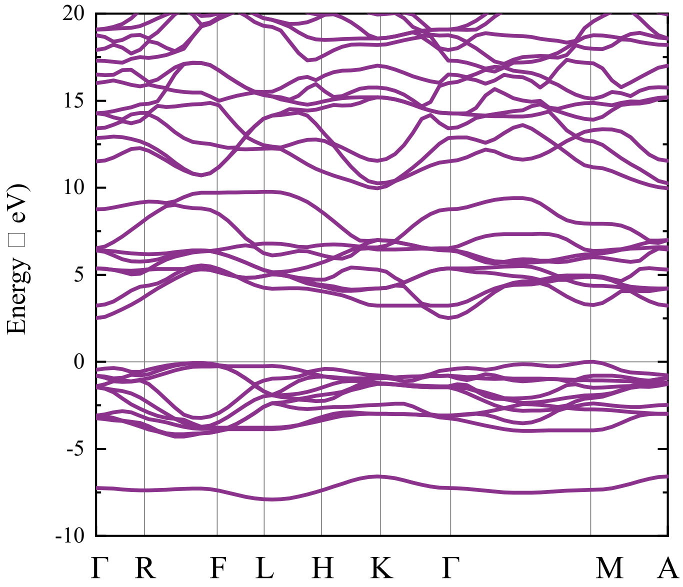

FigS7. The electronic structure of the M_B-Cm phase. The abscissa is the high symmetry point located in the first Brillouin zone. Their local coordinates are (0, 0, 0), (0, ζ, 0), (ζ, ζ, 0), (ζ, 0, 0), (ζ, ζ, ζ), (0, 0, ζ), (0, ζ, ζ), (ζ, 0, ζ) respectively.

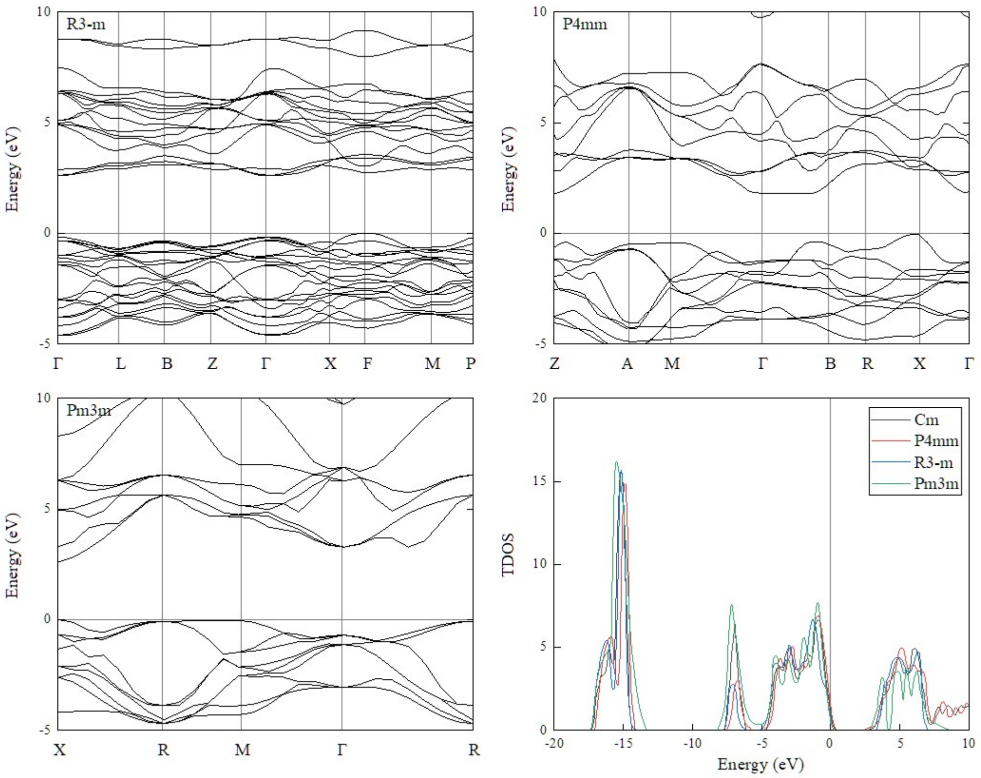

FigS8. The electronic structure and TDOS of the different PZT phases.

	Cm(M_B)	Pm3m	P4mm	R3-m
Ti-O_1	0.0406	0.0427	0.0421	0.0417
Ti-O_2	0.0395		0.0417	0.0421
Zr-O_1	0.0412	0.0431	0.0429	0.0435
Ti-O_2	0.0409		0.0425	0.0436

TableS3. The -COHP of the different PZT phases. Due to the limitations of the VCA, these calculations were based on 2×4×4 supercells, where the atomic distribution belongs to the T_d point group, representing a crystal with a Zr/Ti ratio of 1.

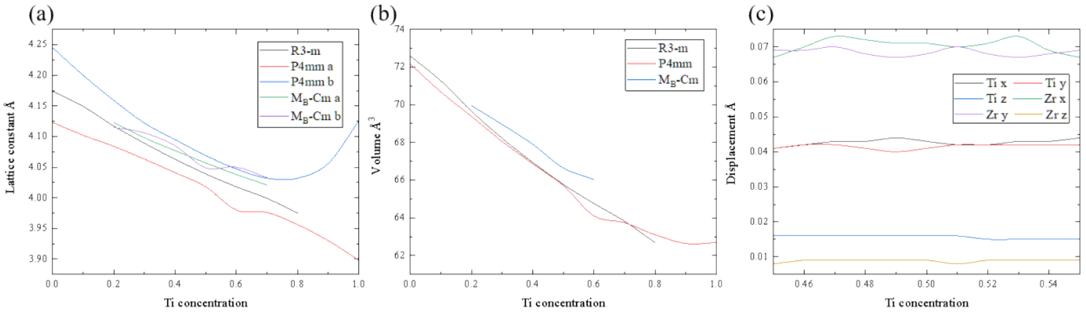

FigS9. The lattice constant (a), volume (b) of three ferroelectric PZT phases; the displacement of central atoms in Cm phase.
